# A Rigid Spine at Risk: Chalk-Stick Fracture With Dural Sac Entrapment After Ground-Level Fall

**DOI:** 10.7759/cureus.113367

**Published:** 2026-07-25

**Authors:** Dylon J Wetzel, Lyndsey N Nguyen, Gregory L Wrubel

**Affiliations:** 1 Medical School, Florida State University College of Medicine, Tallahassee, USA; 2 Radiology, AdventHealth Orlando, Orlando, USA; 3 Neuroradiology, AdventHealth Orlando, Orlando, USA

**Keywords:** ankylosing spondylitis, chalk-stick fracture, computed tomography, dural sac entrapment, low-energy fall, magnetic resonance imaging, nerve root entrapment, spinal subarachnoid hemorrhage, spinal trauma, three-column fracture

## Abstract

Ankylosing spondylitis predisposes patients to unstable spinal fractures following low-energy trauma because of progressive spinal ankylosis and loss of flexibility. These fractures may be difficult to recognize clinically and can be associated with delayed neurologic complications. We present the case of a 65-year-old male patient with ankylosing spondylitis and severe kyphosis who developed progressive left lower extremity pain, weakness, and numbness five days after a ground-level fall. Computed tomography (CT) of the lumbar spine revealed marked widening of the L1-L2 intervertebral disc space with a transverse fracture extending through the ankylosed spine and posterior elements. Magnetic resonance imaging (MRI) further characterized the injury, demonstrating disruption of the anterior and posterior longitudinal ligaments, disc-space widening, posterior element involvement, and entrapment of the anterior dural sac and ventral nerve roots within the fracture defect. Associated intradural hemorrhagic products, nerve root clumping, and paraspinal soft tissue edema were also identified. This case stresses the diagnostic difficulty of spinal trauma in ankylosing spondylitis and accentuates the complementary roles of CT and MRI in evaluating unstable chalk-stick fractures. Although CT readily identifies osseous injury, MRI can reveal clinically significant soft-tissue and neural element involvement that may not be fully appreciated on CT alone. Early recognition of these injuries is essential to reduce the risk of delayed neurologic deterioration and guide appropriate management.

## Introduction

Ankylosing spondylitis (AS) is a chronic inflammatory spondyloarthropathy, a group of inflammatory diseases that primarily affect the axial skeleton (spine and sacroiliac joints). It is characterized by progressive ossification (abnormal bone formation) of the spinal ligaments, intervertebral discs, and facet joints, resulting in a rigid, brittle spinal column. This loss of spinal flexibility predisposes patients to unstable fractures even after low-energy mechanisms of injury. Due to the altered biomechanics of the ankylosed spine, these fractures frequently involve all three spinal columns (the anterior, middle, and posterior structural components of the spine), thereby rendering them highly unstable [1]. Consequently, they carry a substantial risk of neurologic injury and poor clinical outcomes [2].

Chalk-stick fractures are transverse fractures that occur through an ankylosed spine and are classically associated with AS. These injuries may be difficult to recognize on initial clinical evaluation, particularly when symptoms are subtle, or trauma is ostensibly minor [3]. As such, delayed diagnosis has been associated with progressive neurologic deficits and increased morbidity [4].

We present a case of a chalk-stick fracture in a patient with advanced AS following a ground-level fall. Multimodality imaging demonstrated an unstable three-column injury with entrapment of the anterior dural sac and ventral nerve roots within the fracture defect. This case stresses the criticality of maintaining a high degree of suspicion for unstable spinal injury in patients with AS and demonstrates the complementary roles of computed tomography (CT) and magnetic resonance imaging (MRI) in characterizing osseous, ligamentous, and neural element involvement.

## Case presentation

A 65-year-old male patient presented to the emergency department with progressive left lower extremity pain, weakness, and numbness following a ground-level fall. His past medical history was significant for ankylosing spondylitis, severe kyphosis status post C4-T8 fusion, deep vein thrombosis, chronic kidney disease, hyperlipidemia, and bipolar disorder. He was receiving apixaban for anticoagulation because of his history of deep vein thrombosis. Five days prior to presentation, the patient fell backward while walking to the bathroom and landed on his back. Initial evaluation following the injury was limited to a CT examination of the head because of anticoagulation therapy and did not include spinal imaging.

The patient subsequently developed worsening left leg pain radiating to the dorsum of the foot, accompanied by progressive weakness and numbness extending from the left thigh to the dorsum of the foot. He reported no additional trauma, syncope, fever, chills, chest pain, dyspnea, abdominal pain, or urinary symptoms.

**Figure 1:**
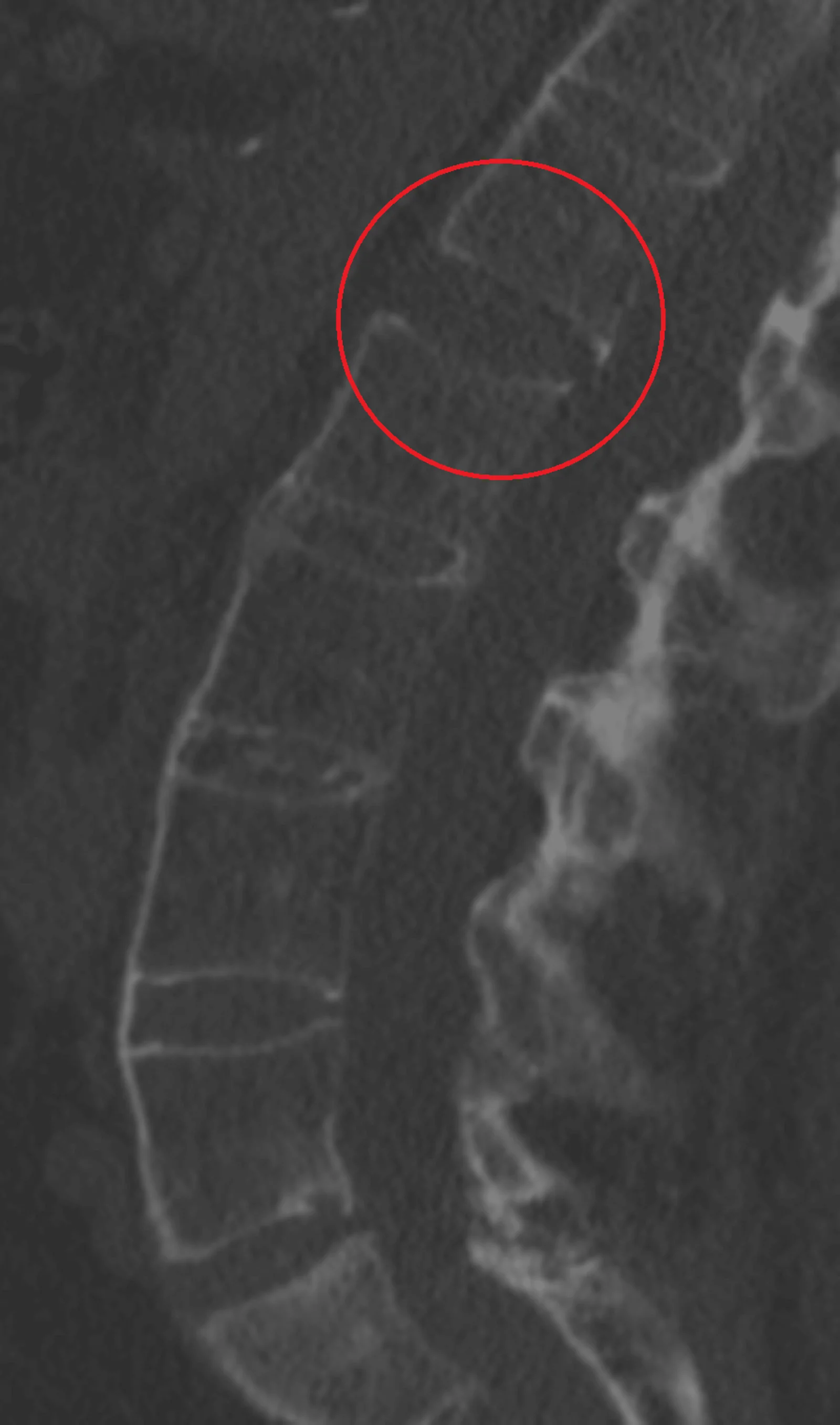
Sagittal CT image demonstrating marked widening of the L1-L2 intervertebral disc space with a transverse fracture extending through the ankylosed spine.

Physical examination demonstrated tenderness of the thoracolumbar spine, left lateral hip, and anterior left thigh. Neurologic examination revealed weakness and sensory deficits involving the left lower extremity. Lower extremity edema was not present. A CT of the lumbar spine revealed marked widening of the L1-L2 intervertebral disc space with an associated transverse fracture extending through the posterior elements in the setting of ankylosing spondylitis (Figure 1).

**Figure 2:**
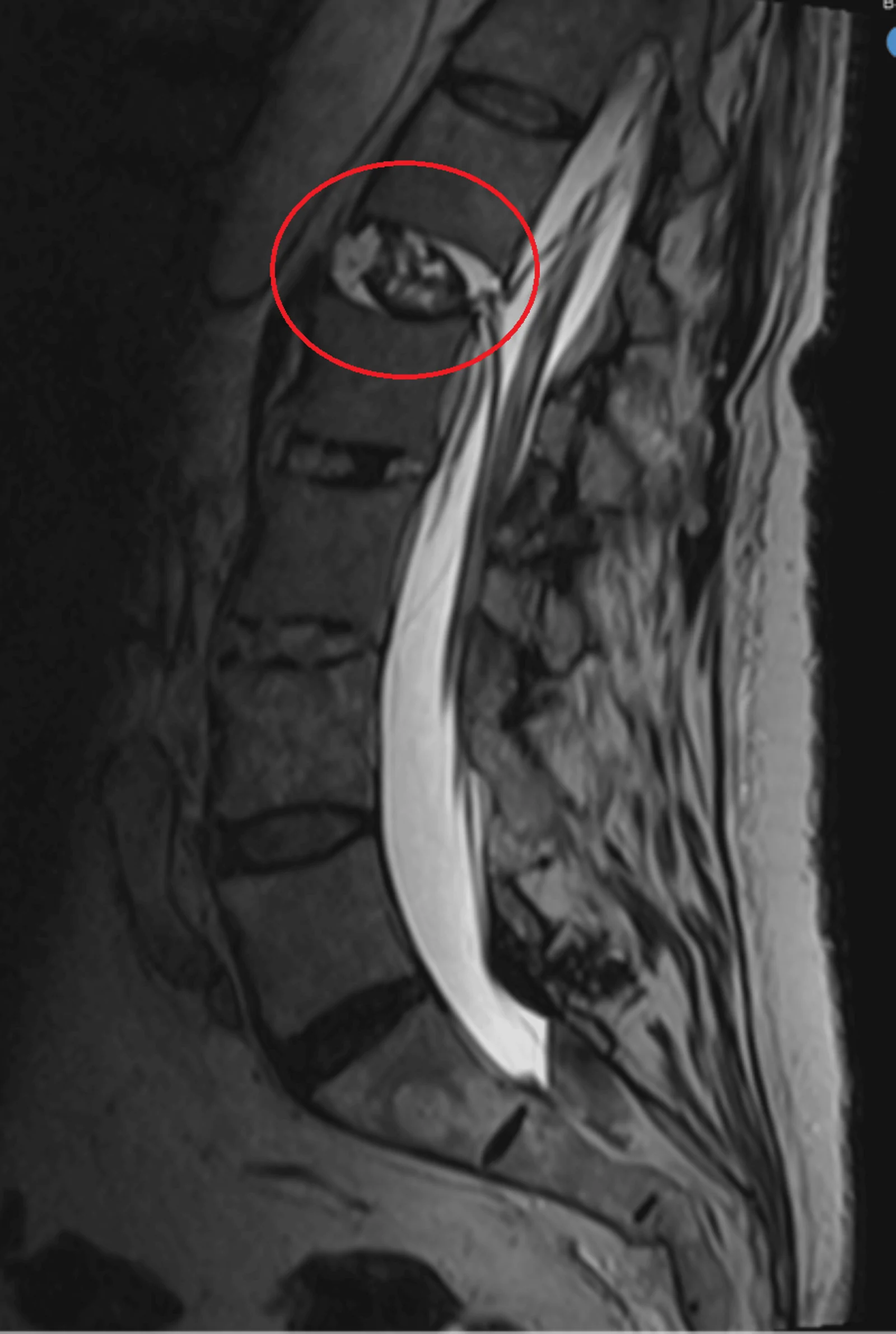
Sagittal T2-weighted MRI demonstrates disruption of the anterior and posterior longitudinal ligaments with entrapment of the anterior dural sac and ventral nerve roots within the fracture defect.

**Figure 3:**
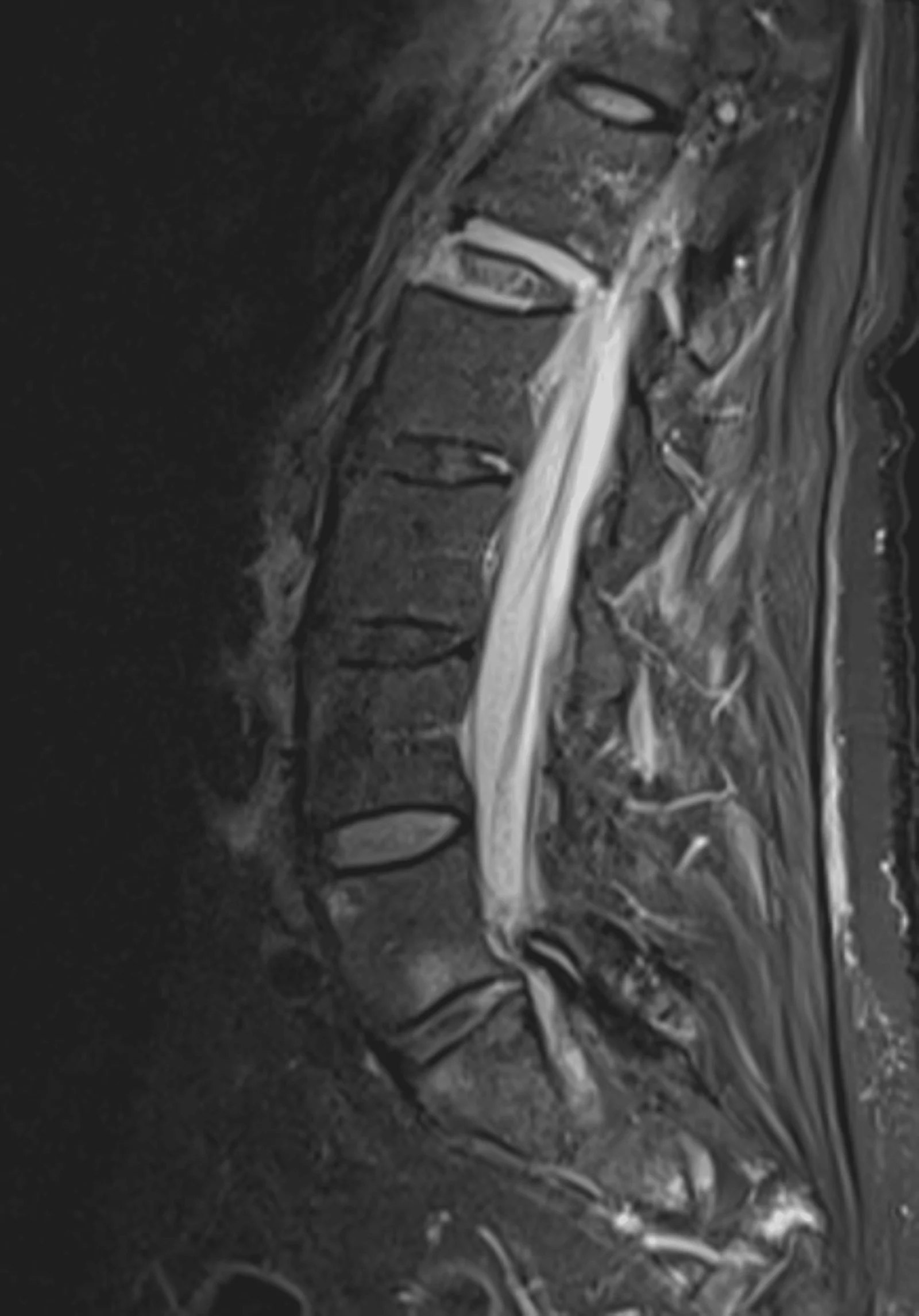
Sagittal STIR MRI demonstrates associated marrow edema and paraspinal soft-tissue injury. STIR, Short Tau Inversion Recovery.

Given the injury's unstable appearance, magnetic resonance imaging of the lumbar spine was subsequently obtained. The MRI demonstrated a three-column chalk-stick fracture with disruption of the anterior and posterior longitudinal ligaments, bilateral laminae, facet joints, and interspinous ligament complex. Additional findings included entrapment of the anterior dural sac and ventral nerve roots within the fracture defect, anterior displacement and clumping of lumbosacral nerve roots, intradural hemorrhagic products with suspected spinal subarachnoid hemorrhage, and associated paraspinal and psoas muscle edema (Figures 2, 3).

Given the imaging findings of an unstable three-column fracture and the patient's persistent neurologic deficits, including left foot drop and bilateral lower extremity weakness, both the neurosurgery and orthopedic surgery teams were consulted. Following their multidisciplinary evaluation, operative stabilization was recommended. The patient subsequently underwent posterior instrumented fusion with posterolateral arthrodesis (surgical joint fusion) from T11 through L4 on post-transfer hospital day 1. Intraoperative CT-guided neuronavigation was used for pedicle screw placement, and a dual-rod construct spanning three levels above and below the fracture was placed to improve construct stability. Neuromonitoring remained stable throughout the procedure, estimated blood loss was 100 mL, and no intraoperative complications occurred.

Postoperatively, the patient was managed with serial neurologic examinations, a thoracolumbosacral orthosis (TLSO) brace while out of bed, fall precautions, and multimodal analgesia. Apixaban was held perioperatively to reduce the risk of surgical bleeding and transitioned to heparin for deep vein thrombosis prophylaxis, with plans to resume apixaban after neurosurgical clearance. On postoperative day 1, he developed confusion and altered mental status consistent with metabolic encephalopathy. Workup later revealed hyperammonemia, and his encephalopathy improved following treatment with lactulose and rifaximin. His postoperative course was also complicated by intermittent hypoxemia requiring supplemental oxygen. Thereafter, physical and occupational therapy evaluations demonstrated severe functional impairment requiring total assistance with transfers and activities of daily living. As such, he was discharged with home health services, durable medical equipment, and outpatient neurosurgical follow-up.

At the subsequent neurology follow-up, the patient remained significantly functionally impaired and required skilled nursing care. His clinical course was further complicated by dysphagia requiring percutaneous endoscopic gastrostomy tube placement, postoperative surgical site infection, urinary tract infection, and progressive cognitive decline related to parkinsonism (Table 1).

**Table 1:** Timeline of clinical presentation, diagnostic evaluation, therapeutic intervention, and follow-up.

Time point	Clinical event
Day 0	Ground-level fall followed by initial evaluation with CT head only; spinal imaging was not performed.
Days 1–5	Progressive left lower extremity pain, weakness, and numbness.
Day 5	Presented to the emergency department with progressive neurologic symptoms. CT and MRI established the diagnosis of an unstable L1-L2 chalk-stick fracture.
Hospital Day 1	Neurosurgical and orthopedic evaluation with recommendation for operative stabilization.
Hospital Day 5	Posterior T11-L4 instrumented fusion with posterolateral arthrodesis performed.
Postoperative Day 1 (Hospital Day 6)	Developed confusion and altered mental status consistent with metabolic encephalopathy.
Postoperative Days 2–4	Hyperammonemia identified; treated with lactulose and rifaximin with clinical improvement.
Hospital Day 12	Discharged with home health services, durable medical equipment, and outpatient follow-up.
~2 Months Postoperatively	Neurology follow-up demonstrated persistent functional impairment requiring skilled nursing care.

## Discussion

Spinal fractures in patients with ankylosing spondylitis present a unique diagnostic challenge because the rigid, ankylosed spine is susceptible to unstable injuries following relatively minor trauma. Progressive ossification of spinal ligaments, intervertebral discs, and facet joints alters normal spinal biomechanics, causing the spine to function as a long lever arm. As a result, even low-energy mechanisms such as ground-level falls can produce highly unstable three-column injuries that place patients at substantial risk for neurologic compromise [1,2].

Delayed diagnosis remains an important concern in this patient population. Clinical symptoms may at first seem disproportionate to the severity of the underlying injury, particularly when trauma is apparently minor, and no immediate neurologic deficits are present [5,6]. In the present case, the patient initially underwent evaluation following a ground-level fall but did not receive spinal imaging until the development of progressive neurologic symptoms several days later. This delay illustrates the criticality of maintaining a high level of suspicion for spinal injury in patients with known AS, regardless of the apparent severity of the inciting event.

Imaging plays a key role in the diagnosis and management of these fractures. CT readily identified the unstable fracture pattern in this patient, whereas MRI provided more thorough characterization by demonstrating disruption of the spinal ligaments, associated soft-tissue injury, and involvement of adjacent neural structures that were not fully appreciated on CT [7,8]. These additional findings further characterized the extent of three-column instability and supported the decision to pursue operative stabilization, portraying how CT and MRI provide complementary information when evaluating spinal trauma in patients with ankylosing spondylitis. Although unstable chalk-stick fractures in AS are well described, the degree of neural involvement demonstrated in this case, including dural sac and ventral nerve root entrapment, was particularly notable and illustrates the value of MRI when neurologic symptoms are present or progressive.

This case emphasizes that advanced imaging should complement, rather than replace, CT when evaluating patients with ankylosing spondylitis who develop neurologic symptoms after trauma. In this case, CT and MRI provided sufficient information for diagnosis and operative planning, underscoring their complementary roles in characterizing osseous, soft-tissue, and neural injury. While CT continues to be indispensable for identifying unstable osseous injury, MRI can detect clinically important soft-tissue and neural involvement that may influence surgical planning and help reduce the risk of delayed neurologic deterioration [4,7,8].

## Conclusions

Patients with ankylosing spondylitis are at increased risk for unstable chalk-stick fractures following low-energy trauma due to the altered biomechanics of the ankylosed spine. This case illustrates the possibility of delayed presentation and significant neurologic involvement despite an apparently minor mechanism of injury. While CT was key for identifying the fracture, MRI provided clinically essential characterization of ligamentous disruption, dural sac entrapment, ventral nerve root involvement, and associated hemorrhagic findings. A high degree of suspicion and appropriate multimodality imaging are essential for the timely diagnosis of spinal injuries in patients with ankylosing spondylitis, particularly when progressive neurologic symptoms are present. Early recognition of these injuries enables prompt surgical stabilization and multidisciplinary management, which may reduce the risk of further neurologic deterioration.
